# High-performance CeO_2_/rGO hybrid nanostructures as bifunctional electrocatalysts for water-splitting

**DOI:** 10.1039/d5ra09363e

**Published:** 2026-05-01

**Authors:** John Andrews Staline.C, C. Maria Magdalane, Gopal Ramalingam, Saikh Mohammad Wabaidur, Jaya Dwivedi

**Affiliations:** a Department of Chemistry Banasthali Vidyapith Rajasthan-304022 India jayadwivedi@yahoo.co.in; b Department of Chemistry, St. Xavier's College (Autonomous), Affiliated to Manonmaniam Sundaranar University Palayamkottai, Abishekapatti Tirunelveli-627012 India; c Quantum Materials Research Lab (QMRL), Department of Nanoscience and Technology, Alagappa University Karaikudi-630003 India; d Chemistry Department, College of Science, King Saud University Riyadh 11451 Saudi Arabia

## Abstract

Hydrogen-evolving material based on two-dimensional reduced graphene oxide with a rare-earth metal oxide such as ceria is relevant for water-splitting devices owing to greater electrocatalytic activity. Herein, a sustainable one-pot hydrothermal synthesis method was chosen to prepare a CeO_2_/rGO nanostructure. The XRD pattern of CeO_2_/rGO validated the intense peak at a 2*θ* value of 26.98°, revealing the formation of CeO_2_ in the graphene layer in the CeO_2_/rGO nanostructure. PL spectra showed a reduction in intensity for the CeO_2_/rGO composite being indicative of strong interfacial interactions between CeO_2_ nanoparticles and rGO. UV-visible spectra suggested higher absorption at 248 and 292 nm for the CeO_2_/rGO composite because of the immobilization of rGO. FESEM of the CeO_2_/rGO nanostructure disclosed that ceria was evenly aligned on rGO sheets around 20–30 nm in size and mapping analysis confirmed a more uniform distribution of elements. HR-TEM of CeO_2_/rGO demonstrated the agglomerated carbon material surrounding CeO_2_ with an average size of 27 nm with the formation of spherical CeO_2_ nanoparticles. The CeO_2_/rGO nanostructure had a Tafel slope of ∼168 mV dec^−1^, which was comparatively smaller than that of CeO_2_, rGO, and the bare NF electrode. The prepared nanostructure demonstrated an overpotential of 260 mV to attain a current density of −50 mA cm^−2^, thereby establishing its superior catalytic activity towards water-splitting.

## Abstract 2

Hydrogen-evolving material based on two-dimensional reduced graphene oxide with a rare-Earth metal oxide such as ceria is relevant for water-splitting devices owing to greater electrocatalytic activity.

## Graphical abstract

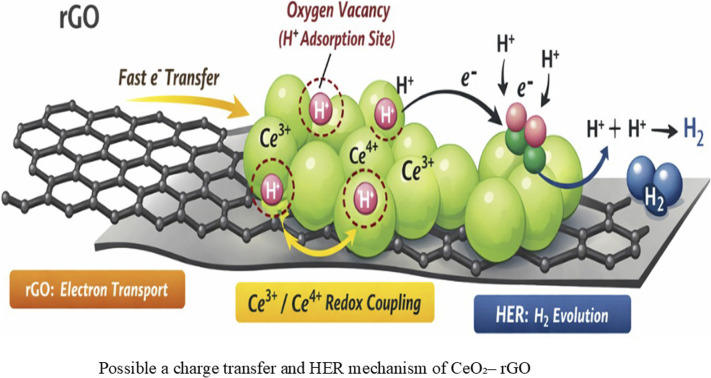

## 1 Introduction

Global energy demand has been rising rapidly over the past century, with projections estimating an increase from 16 terawatts in 2010 to 30 terawatts by 2021.^1^ Currently, the conventional energy sources of coal, petroleum, and natural gas account for 79.5% of total energy consumption, while renewable sources, such as hydroelectric, windmills, bio-power, and solar power, contribute barely 20.5%. This heavy reliance on non-renewable energy has led to destruction and significant environmental challenges.^2^

Molecular hydrogen (H_2_) presents a favorable alternative fuel because of its high energy density and potential for supporting low-carbon energy systems. However, most H_2_ is generated through vapour-reforming of fossil fuels, an inefficient action that consumes fossil resources and releases CO_2_.^3^ To make the hydrogen economy viable, there is a critical need for a clean, renewable, and efficient method of hydrogen production.

The electrolysis of water is recognized as an efficient, and carbon-neutral technique for generating H_2_ and O_2_, making it a key substitute method that relies on fossil fuels. Recent research has shifted towards the development of advanced electrocatalysts using more affordable, Earth-abundant nanostructures to enhance the efficiency of the hydrogen evolution reaction (HER).

At the moment, platinum group metals and noble metal oxides (such as RuO_2_ and IrO_2_) are widely utilized as state-of-the-art electrocatalysts for the HER and oxygen evolution process (OER). Nevertheless, the disadvantages of noble metal electrocatalysts, such as their high price, small storage capacity, and unstable nature, have severely limited their broad application in real-world applications.^4^ Therefore, investigating non-noble metal electrocatalysts with plentiful reserves, comparatively low cost, and high efficiency is extremely difficult yet has significant practical value. Recently, scientists have spent an abundance of time and energy investigating cost-effective, efficient electrocatalysts, particularly transition metals such as M–C^2−2^, –O^2−^, –N^3−^, –S^2−^, –P^3−^, and –Se^2−^. Heterojunction nanostructure materials have been focused upon for attaining good, stable and promising results.^5–7^ Among these, the nanostructures of metal oxides (MO) and reduced graphene oxide (rGO) have emerged as a sustainable strategy.^8–16^ These rGO-metal oxide nanostructures have gained attention due to their versatile applications. Their unique properties make them ideal candidates for advancing sustainable hydrogen evolution technologies, including photocatalytic dye degradation,^17^ supercapacitors,^18^ electrocatalysts,^19^ energy-related technologies,^20–24^ and batteries.^25^ For example, composites of Mo, Mn, and W can improve the water-splitting performance by modifying the electronic structure of basic catalysts. However, the catalytic efficacy and stability of these electrocatalysts are inadequate due to the problem of acid corrosion; they continue to exhibit poor stability and a relatively high HER overpotential. For instance, Cr-doped NiFe_2_O_4_ nanosheets exhibit high current density bifunctional water-splitting, while recent doped ceramic electrocatalysts reported in the literature also show significant HER and OER activity in alkaline media, emphasizing the role of structural and defect engineering in modulating active sites and charge transfer.^26–28^

Despite considerable advances in the development of bifunctional electrocatalysts for overall water-splitting through defect modulation, elemental doping, and heterostructure engineering, several crucial issues need to be addressed. The scalability and practical applicability of many recently reported systems are limited by their reliance on intricate multi-step syntheses, costly transition-metal dopants, or high activity only at low current densities. Furthermore, there is a lack in mechanistic importance of interfacial charge transfer and oxygen vacancy modulation in rare-earth oxide/carbon hybrids, especially in alkaline environments. In particular, sustainable CeO_2_-based bifunctional electrocatalysts supported on conductive carbon frameworks are rare, despite the promising redox flexibility of CeO_2_ and its capacity to produce oxygen vacancies. It has not been thoroughly investigated how CeO_2_–rGO interfacial coupling affects charge-transfer kinetics, electrochemical surface area, and long-term stability at high current densities. The development of a CeO_2_/rGO hybrid electrocatalyst that can concurrently deliver effective HER and OER activity with low overpotentials, quick reaction kinetics, and long-lasting stability in alkaline media is therefore clearly lacking.^27–30^

Ceria-based composites have recently received more attention because of the reversible transformation between Ce^3+^ and Ce^4+^. They have been utilized as good supplementary promoter hybrids to accomplish electrocatalytic activity. Specifically, the CeO_2_/rGO nanostructure stands out for its remarkable high catalytic performance.^30,31^ Motivated by this, we present a simple “greener” method for creating a highly active and effective CeO_2_/rGO electrocatalyst. This was achieved by synthesizing the catalyst from precursors. Because of their enormous specific surface area, structural and functional flexibility, and changeable oxidation state, they have drawn a lot of interest as sacrificial metal oxide composites for water-splitting.

## 2 Materials and methods

Cerium(iii) nitrate hexahydrate (Ce (NO_3_)_3_·6H_2_O, 99.9%), sodium nitrate (NaNO_3_), hydrogen peroxide (H_2_O_2_, 35%), hydrazine hydrate (N_2_H_4_, 99%), potassium permanganate (KMnO_4_, 98.3%), KOH, carbon black (acetylene, 100% compressed, 99.9%), *N*-methyl-2-pyrrolidone (NMP, ≥99.5%), polyvinylidene fluoride (PVDF) and ethanol (≥99.8%) were purchased from Sigma-Aldrich. Nickel foam (NF) was sourced from Indiamart. Chemicals were used without further purification.

### 2.1 Synthesis of rGO

Graphene oxide was produced *via* a modified Hummer's method. Hydrazine monohydrate was utilized as a strong reducing agent to prepare reduced graphene oxide. One gram of GO was dispersed in 100 mL of deionized water and sonicated for uniform dispersion. Then, 10 mL of pure hydrazine hydrate was added dropwise to the solution and refluxed at 100 °C for 24 h. The obtained solid product was washed with DI water several times and pH adjusted to neutral followed by washing with ethanol. The final product was then dried at 60 °C in a vacuum oven for 24 h.

### 2.2 Synthesis of CeO_2_

Two grams of cerium nitrate hexahydrate (Ce(NO_3_)_3_·6H_2_O) were dissolved in 20 mL of water and mixed under ultrasonication for 1 h. Then, 20 mL of starch solution was added dropwise with constant stirring, followed by 5 M NaOH solution until the pH was adjusted to 10. The mixture solution was stirred for 4 h until it was homogenized. Additionally, this mixture solution was poured into a 100-mL autoclave and heated at 120 °C for 3 days. Then, the solution was placed in an autoclave and cooled to room temperature. The precipitate was sequentially washed with distilled water and calcined at 700 °C for 3 h. The final precipitate was cleaned with ethanol and then dried in hot-air oven at 60 °C for 6 h to produce pure yellowish ceria nanoparticles.

### 2.3 Synthesis of ceria/rGO

**Fig. 1:**
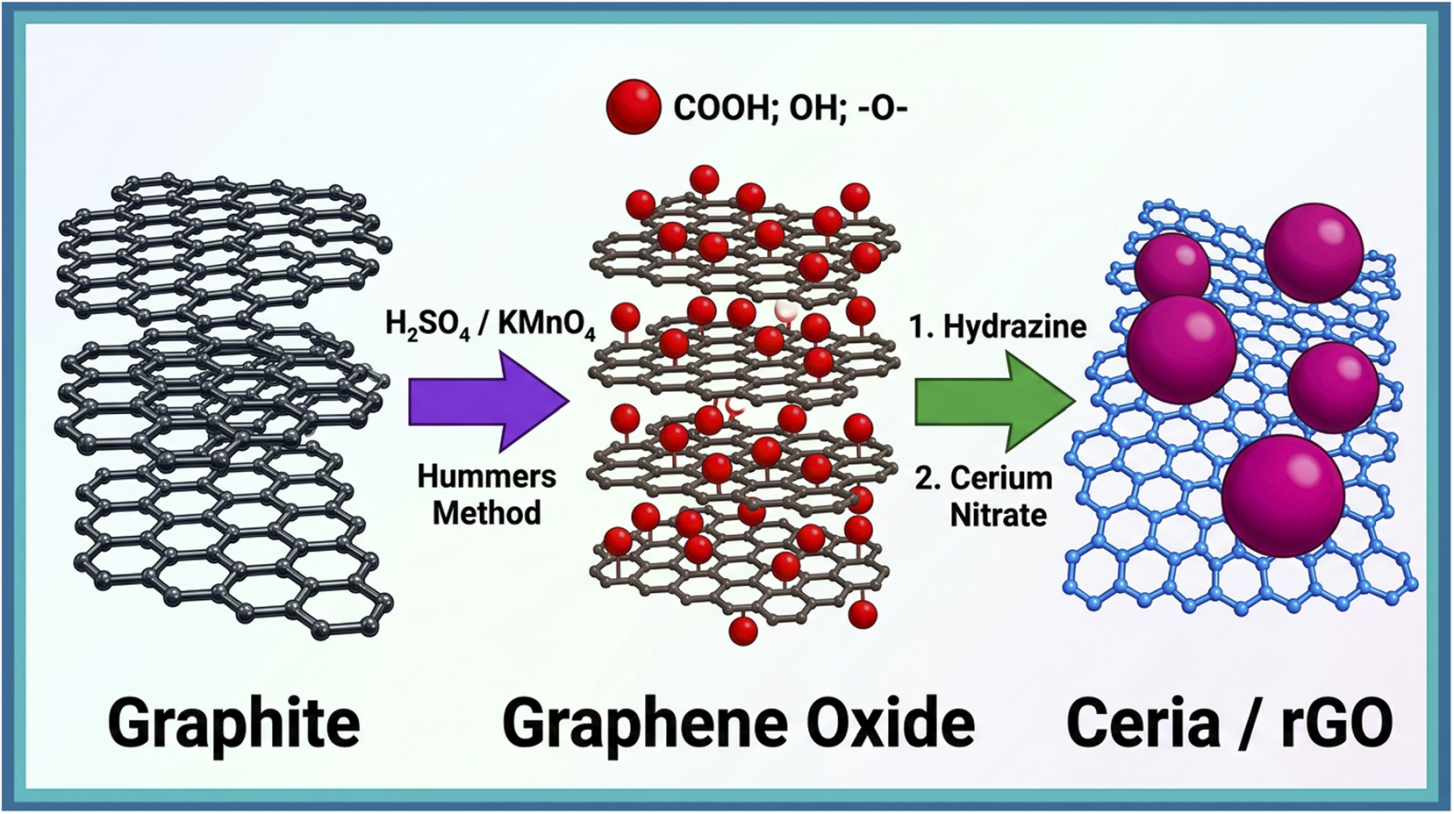
Synthetic procedure of the CeO_2_/rGO nanostructure.

Initially, 50 mg of rGO was added to 50 mL of DI water and ultrasonicated for 30 min. Next, 15 mL of cerium nitrate solution was added and stirred for 2 h. To the above mixture was added 20 mL of starch solution followed by addition of 5 M NaOH solution until the pH was adjusted to 10. Following an ethanol wash, the mixture was placed in a stainless-steel Teflon reactor and heated to 100 °C for 48 h. The resultant precipitate was consecutively washed with distilled water. The impurities in the precipitate were cleaned with ethanol, followed by drying in an air oven at 60 °C for 6 h. The synthetic procedure of CeO_2_/rGO is shown schematically in Fig. 1.

### 2.4 Electrode preparation

NF (1 cm^2^) was first cleaned with HCl, followed by ethanol, and dried to prepare electrodes for electrochemical analysis. Three distinct electrode materials were fabricated by combining 80% by weight of either CeO_2_, rGO, or CeO_2_/rGO nanostructure with 10% carbon black and 10% PVDF. These mixtures were ground into a fine powder, and mixed with NMP to make a sludge. The prepared sludge-like paste was then loaded onto the NF electrode and dried overnight at 80 °C to ensure adhesion and solvent evaporation.

### 2.5 Electrochemical measurements

The electrocatalytic activity of the produced electrodes was assessed using a conventional three-electrode setup and the Biologic SP-300 Potentiostat. The CeO_2_, rGO, or CeO_2_/rGO NF-supported working electrodes, a graphite rod counter electrode, and a Hg/HgO reference electrode were all submerged in 1 M KOH electrolyte. Before measurements, the electrolyte solution was purged with N_2_ gas for 20 min to eliminate dissolved oxygen. A scan rate of 1 mV s^−1^ was used for linear sweep voltammetry (LSV). Using the uncompensated resistance ascertained by electrochemical impedance spectroscopy (EIS), 100% *iR* correction was done to guarantee precise potential readings. After that, all potentials were transformed using the Nernst equation to the reversible hydrogen electrode (RHE) scale^32^ (eqn (1)).

1 *E*_RHE_ = *E*_Hg/HgO_ + (0.059 × pH) + 0.09

The observed voltage was *E*_Hg/HgO_, and 1 M KOH had a pH of 14.

A key kinetic measure that illustrates the connection between the electrochemical rate and overpotential—the potential needed to reach each 10 mA cm^−2^ increase in current density—is the Tafel slope. Plotting the log of the current density (*j*) against the overpotential (*η*) allowed for its calculation from LSV readings. The Tafel equation can be obtained from the linear fitting of the Tafel curve^33^ (eqn (2)).

2 *η* = *b* log *j* + *a*

The active surface area of the catalyst was determined using electrochemical surface area (ESCA). The latter was derived from the double-layer capacitance (*C*_dl_) derived from the plot of the cathodic and anodic sweep current density difference, Δ*j* = *j*_A_ − *j*_C_, against scan rates (*v*) from 10 to 50 mV s^−1^. This equation yields a slope value equal to twice the *C*_dl_^34^ (eqn (3)).

3 Δ*j* = *v*_2_*C*_dl_

where current is denoted by *I*, the Avogadro constant by NA, the Faraday constant by *F*, the number of electrons transported per molecule by *n*_1_, and the number of active sites by *n*_2_. The equation can be used to compute *n*_2_ (ref. 31) (eqn (4)).4
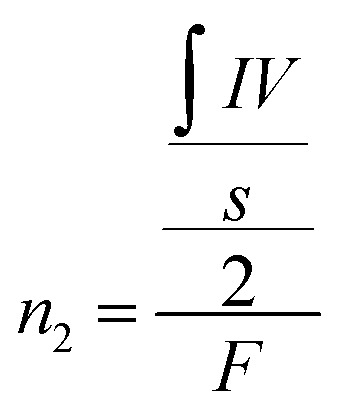
where *S* is the CV scan rate (50 mV s^−1^), *F* is the Faraday constant, and ∫*IV*/*s* is the absolute charge, which is determined by dividing the CV absolute area by the scan rate.

### 2.6 Instrumentation

A Panalytical equipment fitted with a copper K-alpha radiation source was used to investigate the X-ray diffraction (XRD) pattern of manufactured and prepared samples. Using an FE-SEM system (Quanta FEG-250), HR-TEM setup (JEOL-2100), and the EDS system connected to the FE-SEM instrument, the surface properties, micro/nanostructures, and elemental composition of the sample were examined. An Analytik Jena Specord-200 model was used to obtain the absorption spectra in the UV-vis DRS range. An Agilent 8453 UV-visible diode array spectrophotometer was used to record spectra using chloroform as the solvent. An Edinberg FLS 980 spectrometer was used for photoluminescence (PL) observations. The structure was also confirmed using Raman spectrometers (Horiba) and FT-IR spectroscopic techniques. X-ray photoelectron spectroscopy (XPS) with a monochromatic Al K_α_ radiation source (1486.6 eV) using a Thermo Electron ESCALAB 250 system was implemented for additional evaluation of chemical components.

## 3 Result and discussion

### 3.1 XRD analysis

**Fig. 2:**
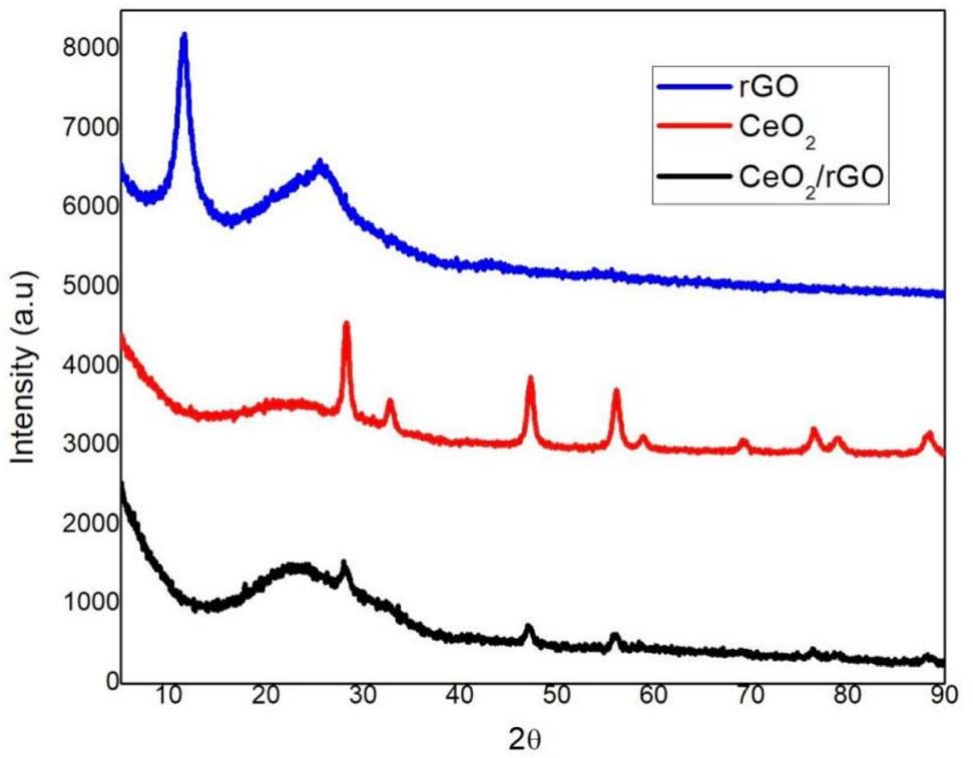
XRD of CeO_2_, rGO and CeO_2_/rGO nanostructures.

The diffraction peaks of pure CeO_2,_ rGO, and CeO_2_/rGO nanostructure are shown in Fig. 2. The powder XRD patterns of the prepared CeO_2_/rGO nanostructure showed very similar diffraction patterns relating to rGO and CeO_2_ in their pure state.^35^ The diffraction peaks were between 25° to 50° and associated with a graphite-like structure. According to the XRD pattern, the diffraction peaks were located at 2*θ* = 28.8, 33.0, 47.4, 56.4, 59.3, 68.7, 76.6, 79.1, and 88.5 and correspondingly allocated as (111), (200), (220), (311), (222), (400), (331), (420), and (422).^36^ Based on data from JCPDS 81-0792, our results confirmed cubic fluorite CeO_2_ with space group Fm-3m and lattice parameters *a* = 5.42 Å and *α* = 90° (52). Furthermore, the presence of CeO_2_ in the graphene layer of the CeO_2_/rGO nanostructure was revealed by the strong peak of the 2*θ* value 26.98° in the pattern of CeO_2_/rGO.^37^ There were no additional peaks for impurities in the sample. Moreover, CeO_2_ nanoparticles were firmly interlaced on rGO sheets.^38,57^

### 3.2 UV-visible absorption spectroscopy

**Fig. 3:**
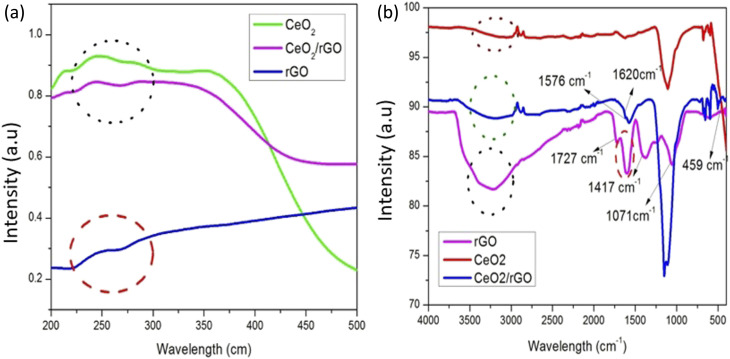
UV-DRS spectrum of the (a) CeO_2_/rGO nanostructure. (b) FT-IR spectrum of the CeO_2_/rGO nanostructure.

The UV-visible absorption spectra of the CeO_2_/rGO nanostructure, rGO, and ceria are displayed in Fig. 3a. The peaks appeared at 248 nm in the CeO_2_ absorption spectra. There was a single peak in the rGO absorption spectra at 242 nm. Furthermore, the absorption maxima of the CeO_2_/rGO nanostructure were located at 248 and 292 nm. The CeO_2_ and CeO_2_/rGO exhibited a prominent UV absorption band. In contrast to CeO_2_, the absorption band in the CeO_2_/rGO nanostructure was red-shifted. The shift in the absorption edge was brought on by the presence of rGO. In comparison with CeO_2_, absorption of the CeO_2_/rGO nanostructure was likewise higher in the visible spectrum. According to their UV-vis spectra, the charge transfer from 2P (O^2−^) to 4f (Ce^4+^) orbitals was connected to the UV zone, which was also associated with the maximum absorbance of CeO_2_. The presence of rGO can contribute to the increase in absorption by altering the process by which electron–hole pairs are formed during irradiation.^39^

### 3.3 FT-IR spectroscopy

FT-IR spectroscopy demonstrated the oxygen-containing functional groups in the synthesized samples shown in Fig. 3b. For rGO, a broad peak with low intensity at 3325 cm^−1^ denoted the –OH group. C=O stretching vibrations were caused by the peak at 1760 cm^−1^.^36^ The peak of the carboxylic group at 1417 cm^−1^ showed the stretching of C–O and O–H bonds. The strong binding energy shown by the epoxy-group peak at 1107 cm^−1^ necessitated high-temperature removal.^40^ The Ce–O vibration band was at 459 cm^−1^ in CeO_2_.^41^ Additionally, elimination of oxygen-containing functional groups was indicated by the disappearance of the absorption band at 1735 cm^−1^ in the FT-IR spectra of CeO_2_/rGO. The distinctive bands of graphene were represented by the bands at 1624 cm^−1^ and 1576 cm^−1^ in the CeO_2_/rGO nanostructure. Furthermore, the band at 489 cm^−1^ illustrated the interaction between CeO_2_ and rGO.

### 3.4 SEM and EDX studies

**Fig. 4:**
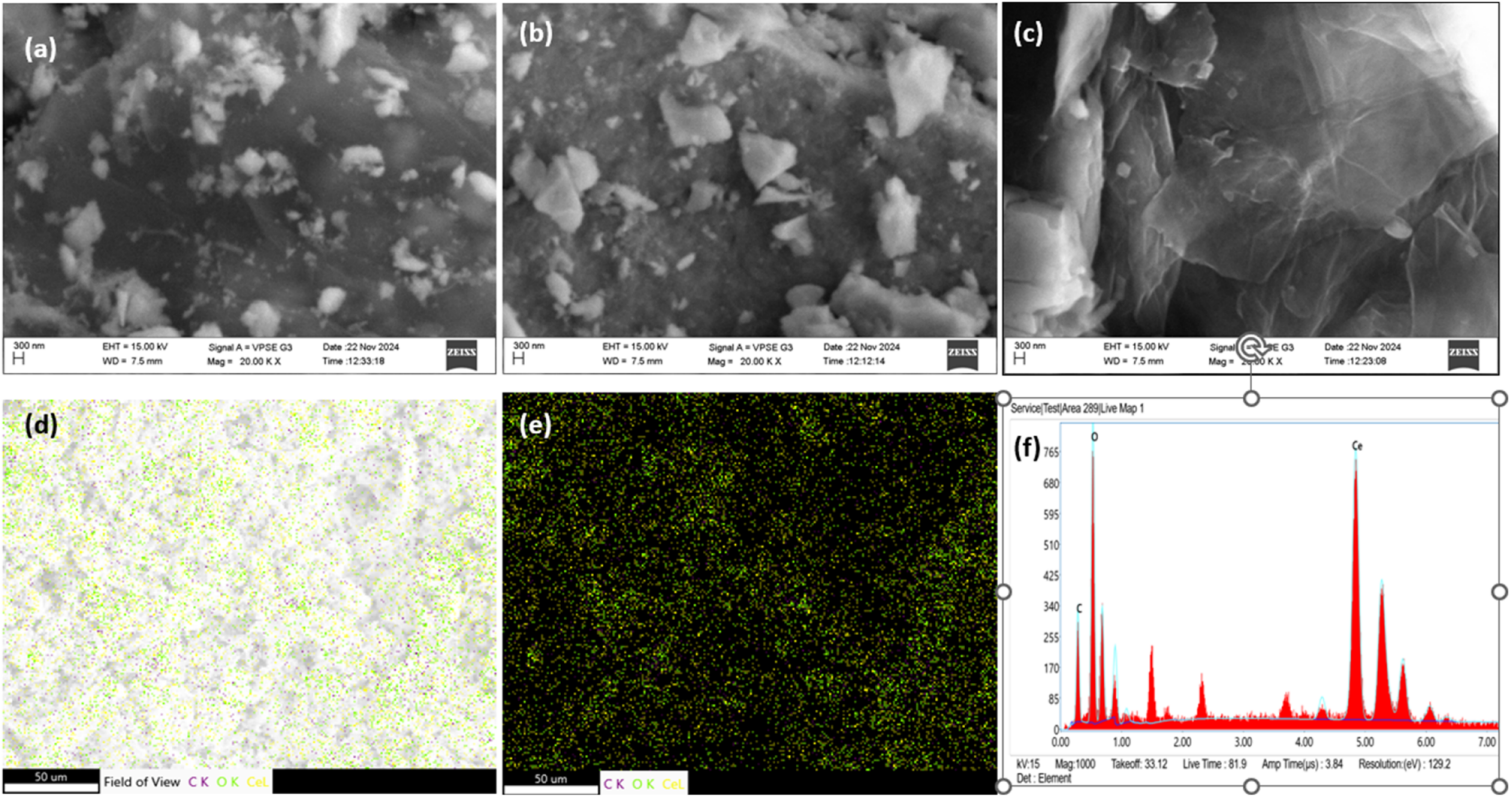
SEM images of (a) pure CeO_2,_ (b) rGO (c) CeO_2_/rGO. (d and e) Elemental mapping and (f) EDX spectrum of CeO_2_/rGO.

The surface morphology of CeO_2_, rGO, and CeO_2_/rGO nanostructure were analyzed using a scanning electron microscope (Fig. 4). All samples exhibited a 3D porous hierarchical structure composed of sheets. The image of CeO_2_ in Fig. 4a demonstrated a rough surface. Moreover, the image of rGO represented in Fig. 4b revealed a 3D-folded, crumbled-layer shape, which facilitates surface porosity and enables effective electron transport.^42^ Moreover, in the CeO_2_/rGO nanostructure, CeO_2_ was identically dispersed on rGO sheets with an average size of 20–30 nm. Fig. 4c shows the rGO surface was beaded with ceria, which was not well distributed. Therefore, they showed high agglomeration. Hence, the excess quantity led to accumulation, and resulted in a change of morphology due to the coordination of the cerium metal ions in the sheets.

**Fig. 5:**
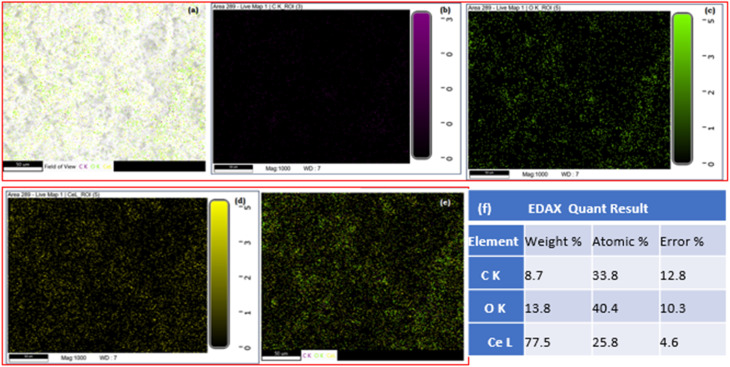
EDX mapping. (a) Field view. (b) Carbon, (c) oxygen. (d) Cerium, (e) all elements. (f) EDAX.

Furthermore, elemental mapping confirmed the existence of cerium, oxygen, and carbon elements in the CeO_2_/rGO nanostructure (Fig. 4). EDX indicated that the nanostructure contained cerium (Ce) with a weight percentage of 77.5%, carbon (C) at 13.8%, and oxygen (O) at 8.7% (Fig. 4f). Fig. 5a–d provided evidence for the existence of Ce, C, and O, thereby confirming the existence of the CeO_2_/rGO nanostructure in mapping.^43^

### 3.5 Transmission electron microscopy analysis

**Fig. 6:**
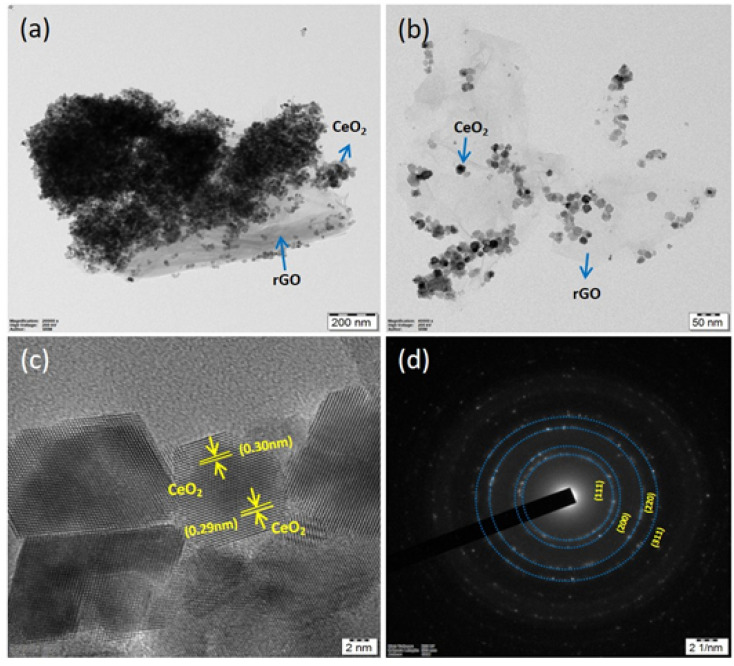
TEM (a–c) and SAED pattern (d) of the CeO_2_/rGO nanostructure.

HR-TEM confirmed the structure of the CeO_2_/rGO nanostructure (Fig. 6). The agglomeration of CeO_2_ had a characteristic layered morphology (Fig. 6a). Furthermore, high magnification of the CeO_2_/rGO nanostructure more clearly demonstrated the agglomerated carbon material surrounding CeO_2_ with an average size of 27 nm (Fig. 6b) and that CeO_2_ was evenly anchored on rGO. The crystalline size of CeO_2_/rGO was concurrent with the XRD pattern. The lattice fringes of CeO_2_-rGO with an interlayer distance of 0.30 nm were associated with the (111) plane of ceria (Fig. 6c).^44^ The selected area electron diffraction pattern of CeO_2_/rGO (Fig. 6d) revealed the fringes of the nanostructure exposed the polycrystalline nature of ceria.

### 3.6 PL studies

**Fig. 7:**
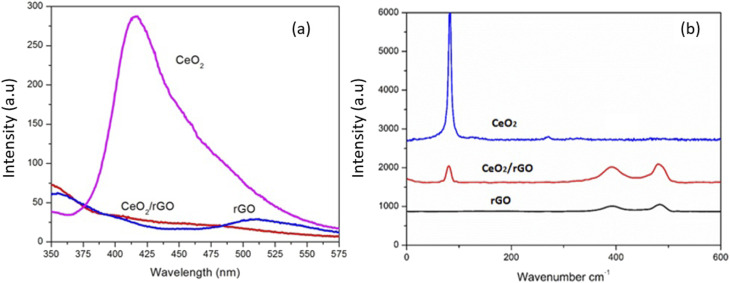
(a) PL spectrum of CeO_2_, rGO and the CeO_2_/rGO nanostructure. (b) FT- Raman spectrum of CeO_2_, rGO and the CeO_2_/rGO nanostructure.

Fig. 7a displays the room-temperature PL spectrum of CeO_2_, rGO, and the prepared CeO_2_/rGO nanostructure under 350-nm excitation. The emission peaks in the range 350 to 500 nm were associated with defect states, particularly oxygen vacancies, suggesting a coupling between Ce 4f and O 2p orbitals. These defect-related energy levels in CeO_2_ were primarily caused by oxygen vacancies below the CeO_2_ 4f band. At room temperature, electron transitions predominantly occur from the defect states to O 2p levels. The PL characteristics of CeO_2_ in the present study align with previously reported results. CeO_2_ exhibited the highest PL intensity among samples, suggesting a lower probability of electron–hole separation.^45^ However, when CeO_2_ was integrated with rGO, the PL intensity of CeO_2_ decreased significantly, while the PL intensity of rGO increased. This could be explained by the effective transfer of excited electrons from the conduction band (CB) of CeO_2_ to rGO, which benefits from the favorable energy band alignment between CeO_2_ and rGO. Compared with pure CeO_2_ and rGO, the electron–hole recombination time in the CeO_2_/rGO nanocomposite was prolonged. The observed reduction in PL intensity for the CeO_2_/rGO nanostructure was indicative of strong interfacial interactions between CeO_2_ nanoparticles and rGO, which was consistent with TEM observations.^46^

### 3.7 FT-Raman spectroscopy

Fig. 7b displays the Raman spectra of rGO, CeO_2_, and the CeO_2_/rGO nanocomposite. The symmetric vibrational phase in the cubic framework of CeO_2_ was represented through a prominent and strong band for the pure CeO_2_ material at ∼465 cm^−1^ caused by the symmetrical stretching mode of the Ce–O_2_ vibrational unit. This narrow range and strong intensity peaks were due to the remarkable crystallinity of CeO_2_ nanoparticles. CeO_2_/rGO showed a blue shift corresponding to the peak at 461 cm^−1^, suggesting the anchoring of CeO_2_ on rGO. This blue shift resulted from the charge transfer between CeO_2_ and rGO (Fig. 9). This peak showed that CeO_2_ nanoparticles were attached to rGO, with a blue shift to 461 cm^−1^ in the CeO_2_-rGO nanostructure.^47^

On the other hand, the amorphous carbon structure corresponding to the rGO spectrum exhibited weak and wide background peaks within the low-wave number region. CeO_2_/rGO showed a blue shift with the reduction in peak intensity corresponding to the 461 cm^−1^ peak, showing anchoring of CeO_2_ on rGO. This also referred to CeO_2_ and rGO charge transfer.

The prominent peak at 465 cm^−1^ in the CeO_2_ Raman spectrum was caused by the symmetrical stretching mode of the Ce–O vibrational unit. This peak showed that CeO_2_ nanoparticles were attached to rGO, with a blue shift to 461 cm^−1^ in the CeO_2_-rGO nanostructure. This blue shift resulted from the charge transfer between CeO_2_ and rGO (Fig. 7b). At the CeO_2_–rGO interface, these structural changes demonstrated that CeO_2_ nanoparticles and rGO sheets had strong interfacial interactions which could result in the development of oxygen vacancies, lattice distortions, and charge transfer. These modifications corroborated the effective incorporation of CeO_2_ into the rGO architecture and enhanced the electronic conductivity and catalytic efficiency of the nanocomposite.^48^

### 3.8 XPS analysis

**Fig. 8:**
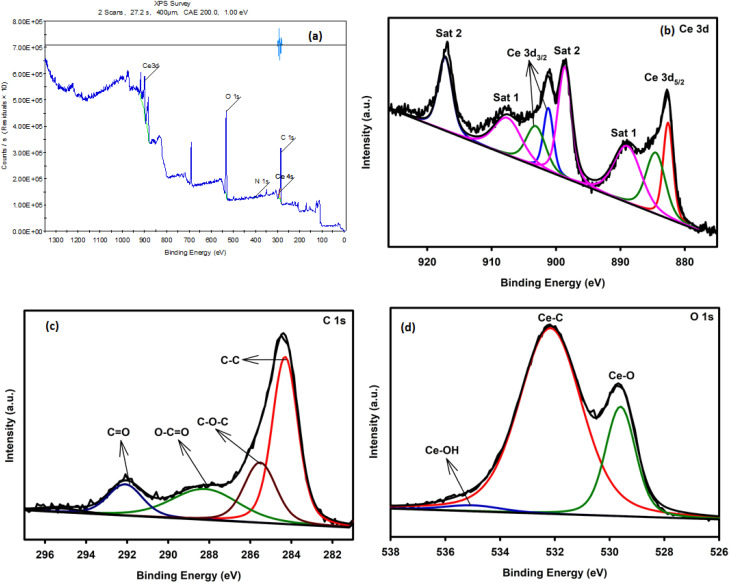
XPS spectra of the CeO_2_/rGO nanostructure.

XPS was used to examine the chemical states of the CeO_2_/rGO heterostructure (Fig. 8). The survey scan of CeO_2_/rGO (Fig. 8a) showed the existence of Ce, O, and C. Ce 3d_3/2_ peaks at 906.78 and 900.96, and Ce 3d_5/2_ peaks at 890.89, 885.89, and 882.28 eV, were associated with Ce^4+^, and peaks at 910.40 eV (Ce 3d_3/2_) and 890.26 eV (Ce 3d_5/2_) were associated with Ce^3+^, all of which suited the Ce 3d spectrum of CeO_2_/rGO shown in Fig. 8b.^45^ The carbonyl C=O bonds, C–O–C, and C–C bonds were responsible for the peaks in the C 1s spectrum (Fig. 8c) of the rGO-CeO_2_ nanostructure that occurred at 291.80, 285.38, and 283.58 eV, respectively. The lattice and adsorbed oxygen were responsible for the peaks at 532.09 and 529.07 eV seen in deconvolution of the O 1s spectrum (Fig. 8d). These findings demonstrated that a GO nanolayer was present on CeO_2_/rGO. Survey scans of CeO_2_ and rGO (Fig. S8.1 and 8.2) are discussed in the SI File.

### 3.9 Hydrogen evolution reaction (HER)

**Fig. 9:**
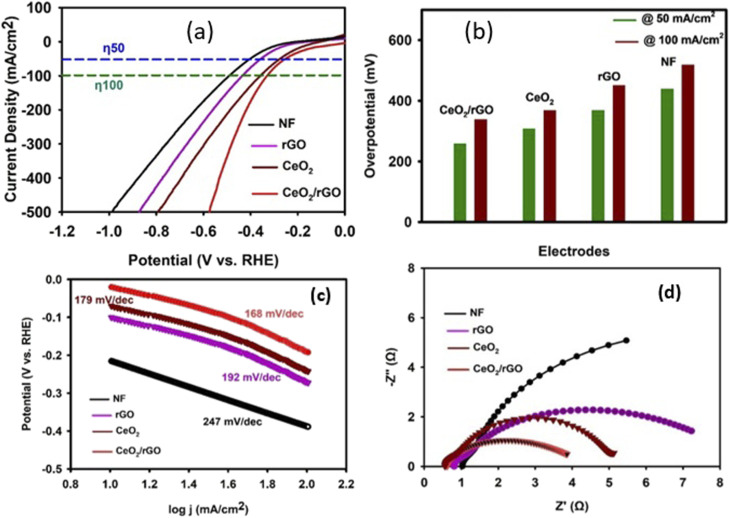
(a) LSV polarization curve, (b) HER overpotential, (c) HER Tafel slope (d) and EIS Nyquist plots of NF, rGO, CeO_2_ and the CeO_2_/rGO nanostructure.

The HER performance of CeO_2_, rGO, or CeO_2_/rGO nanostructure and bare NF electrodes was examined by LSV (Fig. 9a). At a scan rate of 1 mV s^−1^ and a potential range of 0 to −1 V *vs.* RHE were employed in 1 M KOH electrolyte. The CeO_2_/rGO nanostructure electrode performed better and exhibited a lower onset potential of −0.23 V and −0.34 V at 50 mA cm^−2^ and 100 mA cm^−2^*versus* RHE, respectively; CeO_2_, rGO, and NF exhibited values of −0.30 V, −0.36 V and −0.43 V at 50 mA cm^−2^ and −0.36 V, −0.44 V and −0.51 V at 100 mA cm^−2^*versus* RHE, respectively. Hence, the CeO_2_/rGO nanostructure showed a minimized overpotential when compared with the other composites, thereby indicating higher catalytic activity. The LSV results demonstrated the superior HER performance of the CeO_2_/rGO nanostructure compared with its components (CeO_2_, rGO) and the bare NF electrode. This enhanced activity could be attributed to several synergistic factors for the combination of CeO_2_ and rGO, creating a material with a significantly higher surface area and active catalytic sites than the other components. The porous structure of rGO and the presence of defects or oxygen vacancies in CeO_2_ provide abundant sites for hydrogen adsorption, a crucial step in the HER process.^49^ This increased density of active sites facilitates a higher rate of hydrogen evolution. The excellent electrical conductivity of rGO creates a conductive network within the composite. This network enables rapid electron transfer to active sites, lowering the kinetic barrier for the HER and increasing the reaction rate. Essentially, reduced graphene oxide (rGO) functions as an electron conduit, ensuring a rapid transport of electrons for the reduction of protons to hydrogen gas. The intense interaction between CeO_2_ and rGO likely leads to a synergistic effect that optimizes the electronic structure of the composite. This phenomenon can modify the hydrogen adsorption energy, making it more favorable for the HER. Furthermore, this interaction may promote a more efficient HER mechanism, potentially favoring Volmer–Heyrovsky^50^ or Volmer–Tafel^51^ pathways, leading to faster hydrogen generation. The CeO_2_/rGO nanostructure exhibited enhanced HER performance due to an increased number of active sites, improved charge transfer kinetics, and synergistic interactions between CeO_2_ and rGO. This makes it a promising candidate for use in electrocatalysts for hydrogen production.

Fig. 9b provides a clear comparison of the overpotentials required by different electrocatalysts to achieve current densities of −50 mA cm^−2^ and −100 mA cm^−2^, which are important metrics for evaluating the performance of HER catalysts. The CeO_2_/rGO nanostructure exhibited the lowest overpotentials among all tested materials. It required only 260 mV to reach −50 mA cm^−2^ and 350 mV to reach −100 mA cm^−2^, demonstrating its superior catalytic activity for the HER. In comparison, the individual components (CeO_2_ and rGO) and the bare NF electrode (310 mV, 370 mV, and 440 mV at 50 mA cm^−2^, and 370 mV, 450 mV and 520 mV at 100 mA cm^−2^*versus* RHE, respectively) showed significantly higher overpotentials, indicating their lower efficiency in catalyzing the HER. The superior performance of CeO_2_/rGO could be attributed to the synergistic effects between CeO_2_ and rGO. The high surface area of the composite provided abundant active sites for the HER, while the excellent conductivity of rGO facilitated efficient charge transfer. Moreover, the interaction between CeO_2_ and rGO may have optimized the electronic structure and hydrogen adsorption energy, further enhancing the catalytic activity.

The Tafel plot (Fig. 9c) provides valuable insights into the HER kinetics of different electrocatalysts. The Tafel slope, derived from the linear portion of this plot, reflects the rate-determining step in the HER mechanism and how effectively an electrocatalyst facilitates this step. A lower Tafel slope indicates faster HER kinetics and a more efficient catalytic process. The CeO_2_/rGO nanostructure exhibited a Tafel slope of ∼168 mV dec^−1^, which was significantly lower than that of CeO_2_, rGO and bare NF electrode (∼179 mV dec^−1^, ∼192 mV dec^−1^, and ∼247 mV dec^−1^). This lower Tafel slope for CeO_2_/rGO suggested a more favorable HER pathway with faster reaction kinetics. This implied that the CeO_2_/rGO electrocatalyst could achieve higher current densities at lower overpotentials, making it more energy-efficient for hydrogen production. The lower Tafel slope of CeO_2_/rGO could be attributed to several factors, including the conductive network provided by rGO, which facilitates rapid electron transfer to the active sites, accelerating the HER process. The synergistic interaction between CeO_2_ and rGO may optimize the hydrogen adsorption energy on the catalyst surface, leading to a more efficient reaction pathway. The Tafel slope value suggested that the HER on CeO_2_/rGO may proceed through the Volmer–Heyrovsky mechanism, where the rate-determining step is the electrochemical desorption of hydrogen. This mechanism is generally associated with faster kinetics than the Volmer–Tafel mechanism. In conclusion, analysis of the Tafel further confirmed the superior HER performance of the CeO_2_/rGO nanostructure, highlighting its potential as an efficient and cost-effective electrocatalyst for hydrogen production.

Electrochemical impedance spectroscopy (EIS) was investigated to study charge transfer kinetics at the electrode–electrolyte interface. Fig. 9d presents the Nyquist plots obtained for the different electrodes. These plots provide information about the resistance to charge transfer (*R*_ct_), a crucial factor determining the HER kinetics. The Nyquist plot for the CeO_2_/rGO electrode displayed a significantly smaller semicircle diameter compared with that of the other electrodes, indicating a much lower Rct of 3.2 Ω. In contrast, the CeO_2_, rGO, and bare NF electrodes exhibited higher *R*_ct_ values of 4.6 Ω, 7.2 Ω, and 5.1 Ω, respectively. This lower *R*_ct_ for CeO_2_/rGO signified rapid and good electron transfer between the electrode and electrolyte during the HER. This enhanced charge transfer could be attributed to the excellent electrical conductivity of the rGO network within the composite, which facilitated the rapid movement of electrons to the active sites where the HER occurs. The lower Rct observed for the CeO_2_/rGO electrode was consistent with its superior HER performance observed in the LSV, overpotential, and Tafel analyses. Efficient charge transfer is essential for achieving high HER activity because it ensures a rapid supply of electrons to reduce protons to hydrogen gas. EIS data further supported the conclusion that the CeO_2_/rGO nanostructure was a promising electrocatalyst for hydrogen production, owing to its favorable charge transfer kinetics and enhanced HER performance.

**Fig. 10:**
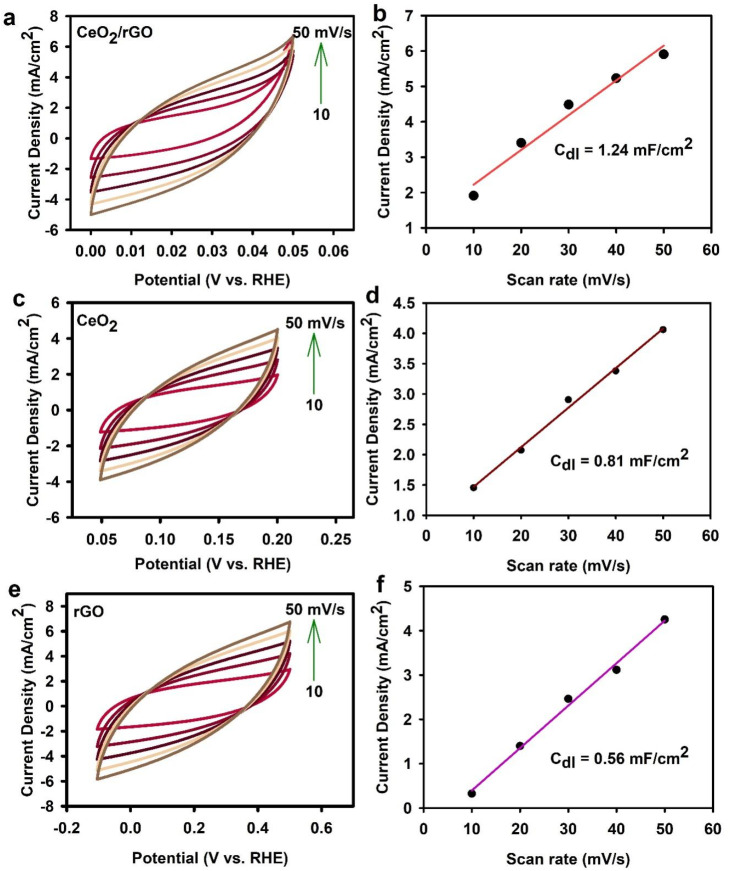
(a–f) Double-layer capacitance (*C*_dl_) slope.

To further assess catalytic activity, the ECSA of the electrodes was evaluated using cyclic voltammetry (CV) in the non-faradaic region (Fig. 10). The double-layer capacitance (*C*_dl_), proportional to the ECSA, was determined by measuring the capacitive current at various scan rates (10 to 50 mV s^−1^). The CeO_2_/rGO electrode exhibited the highest Cdl of 1.24 mF cm^−2^, indicating a larger ECSA than the CeO_2_ (0.81 mF cm^−2^) and rGO (0.56 mF cm^−2^) electrodes. This higher ECSA for CeO_2_/rGO suggested a more significant number of active sites available for the HER, contributing to its enhanced catalytic performance. In part, the increased surface area and enhanced activity of the CeO_2_/rGO nanostructure could be attributed to oxygen vacancies in CeO_2_. These vacancies act as trapping sites for positively charged hydrogen ions (H^+^), facilitating their adsorption and subsequent reduction to hydrogen gas. This effect contributed to the faster reaction kinetics observed for the CeO_2_/rGO electrode. Comprehensive electrochemical characterization, including LSV, overpotential analysis, Tafel slopes, and ECSA, consistently demonstrated the superior HER performance of the CeO_2_/rGO nanocomposite. Its quick reaction kinetics, higher performance, and larger ECSA compared with those of the individual components (CeO_2_ and rGO) and bare NF electrode make it a promising candidate for developing effective and low-cost electrocatalysts for H_2_ generation.

**Fig. 11:**
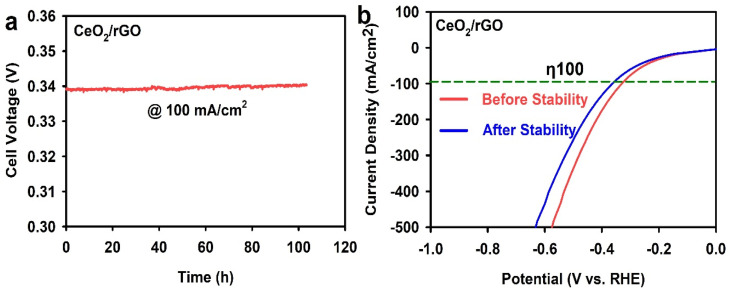
(a) Chronopotentiometry stability test and (b) LSV polarization curve before and after 100 h.

The cyclic stability of the prepared CeO_2_/rGO was assessed using chronopotentiometry, a technique that measures the potential of an electrode over time while maintaining a constant current density. In Fig. 11a, the electrode was subjected to a constant current density of −100 mA cm^−2^ for 100 h. The CeO_2_/rGO electrode demonstrated excellent stability throughout the 100-h test, with only a minor increase in the overpotential required to maintain the desired current density. This stability indicated the robust nature of the electrocatalyst and its ability to withstand prolonged operation without significant degradation in performance. Fig. 11b provides a closer look at the change in potential before and after the stability test. Initially, the electrode required a potential of −0.34 V to achieve a current density of −100 mA cm^−2^. After the 100-h stability test, this potential increased slightly to −0.37 V. This slight increase in overpotential suggested minimal degradation of the electrocatalyst, further highlighting its long-term durability. The excellent stability of the CeO_2_/rGO electrode could be attributed to the good interplay between CeO_2_ and rGO helping maintain the structural integrity of the composite, preventing detachment or aggregation of the active components. CeO_2_ has good corrosion resistance, which helps to protect the electrode from degradation in an alkaline electrolyte, and the rGO network provides a stable and conductive scaffold for CeO_2_ nanoparticles, ensuring efficient charge transport throughout the electrode. The longer stability of an electrocatalyst is critical for its implementation in water-splitting devices. The chronopotentiometry test demonstrated the potential of the CeO_2_/rGO nanostructure for long-term and efficient hydrogen production. Further Table 1 give comparative HER performance clearly indicate non-noble electrocatalysts.

**Table 1:** Comparison of HER performance of our catalyst with that of recently reported non-noble electrocatalysts in alkaline media

Catalyst	Electrolyte	*η*_10_ (mV)	*η*_50_ (mV)	Tafel slope (mV dec^−1^)	Stability test	Reference
CeO_2_/rGO/NF (present work)	1 M KOH	180	260	168	100 h @ −100 mA cm^−2^ (Δ*η* ≈ 30 mV)	Present work
FeNiSe/rGO	1 M KOH	140	—	136.3	—	48
Ru@P-rGO	1 M H_2_SO_4_	268	—	141	12 h @ −10 mA cm^−2^	49
rGO-based BaZnO_2_ composite	1 M KOH	206	—	65	50 h @ −10 mA cm^−2^	50
MoS_2_–rGO/Mo	1 M KOH	—	∼291 mV (for −100 mA cm^−2^)	52	20 h @ −10 mA cm^−2^	51

### 3.10 Oxygen evolution reaction (OER)

In the OER analysis, the rGO, CeO_2_, and CeO_2_/rGO were combined with NMP in a different container to create the working electrodes. Following that, each mixed slurry was applied to a 1 cm^−2^ NF platform and allowed to dry for 5 h at 100 °C. This was important to highlight that the saturated calomel electrode (SCE) and platinum mesh were utilized as reference and counter electrodes, correspondingly. After the rGO, CeO_2_, and CeO_2_/rGO working electrodes had been created, the OER attainment in a 1 M KOH electrolyte was examined using a three-electrode system (VersaSTAT3; Ametek Scientific Instruments). KOH pellets were of standard quality (Sigma Aldrich). Chronopotentiometric measurements were made of the rGO, CeO_2_, and CeO_2_/rGO electrodes at injection current densities ranging from 10 to 100 mA cm^−2^.

During the OER investigation, two crucial variables were noted as the main obstacles that water electrolyzers must overcome. Catalytic intermediate substances formation and their association to active sites constitute the initial mechanism. The second aspect, resulting in slower dynamics and an elevated anodic overpotential, involves the creation of subatomic oxygen atoms through the combining of O* molecules. Comparing CeO_2_/rGO nanostructures to the results of other fabricated samples indicated that the structures had a reduced starting potential for the OER.

**Fig. 12:**
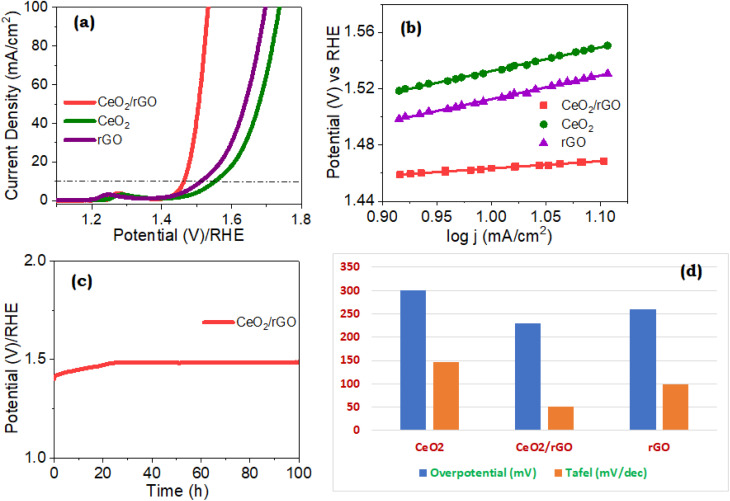
(a) LSV polarization curves, (b) OER Tafel slopes, (c) chronopotentiometry stability test, and (d) comparison of overpotentials and Tafel slopes of the nanostructure.

Fig. 12a shows the overpotential (*η*) of the rGO, CeO_2,_ and CeO_2_/rGO nanostructures specimen on the basic of *iR*-corrected LSV. In comparison with rGO and CeO_2_, the CeO_2_/rGO nanostructures demonstrated higher electrocatalytic performance, attaining a current density of 10 mA cm^−2^ at an OER overpotential of 230 mV, 260 mV, and 300 mV, respectively (Fig. 12a). As illustrated in Fig. 12b, the Tafel slopes reflected the quicker kinetics linked to CeO_2_/rGO nanostructures as revealed by the anodic LSV polarization curves, which showed notable variations at a *v* of 10 mV s^−1^. The improved OER performance of CeO_2_/rGO over rGO and CeO_2_ suggested that the beneficial interaction of multiple elements had a role in their outstanding performance. From a kinetic perspective, the OER features could be further explained using the Tafel slope. A narrower Tafel slope indicates a faster OER kinetic process. CeO_2_/rGO nanostructures showed the shortest Tafel slope among these evaluated catalysts, indicating their kinetic process of increasing the OER. In comparison with CeO_2_, the Tafel slope of CeO_2_/rGO nanostructures was slightly lower. These low Tafel slopes of the material implied that the dominating surface covering determined the overall OER rate.

These results demonstrated the improved electrochemical activity and robustness for CeO_2_/rGO nanostructures by exhibiting the least variation in overpotential and the least decrease in current density. Using an intricate interaction involving several methods, CeO_2_/rGO nanostructures have been illustrated to greatly increase the electrocatalytic activity of the OER in alkaline circumstances. By increasing the number of active sites for the OER, this increased surface area improves catalytic efficiency. Additionally, the electrolyte may be readily introduced in CeO_2_/rGO nanostructures, which also facilitate ion transport and enhance the electrocatalytic activity. OER performance is then enhanced and reaction kinetics are accelerated as an outcome. Enhancing electrocatalytic activity also depends heavily on inherent flaws in the CeO_2_ lattice. Lattice distortions and oxygen vacancies add more active sites and alter the electrical structure of the material. By encouraging the attachment and activation of reactive oxygen species at active sites, these variations improve the suitability for the OER of the material. Low charge transfer resistance is another feature of CeO_2_/rGO nanostructures that suggests effective electron transfer throughout the OER performance. The reaction rates and total electrocatalytic performance are improved by this effective charge carrier transfer. Table 2 demonstrates a detailed comparison of similar CeO_2_/carbon-based composites for deeper understanding. Table 2 reveals that the proposed composite would be a better choice for enhanced electrocatalytic activity.

**Table 2:** Comparison of CeO_2_/carbon-based composites for electrocatalytic activity

Material	Electrolyte	OER *η* (mV)	HER *η* (mV)	Stability	Ref.
CeO_2_-GO	1.0 M KOH	240 @ 10 mA cm^−2^	—	∼10 h stable	52
FexNiy/CeO_2_ on N-doped nanocarbon	1.0 M KOH	240 @ 10 mA cm^−2^	260 @ 50 mA cm^−2^	Overall cell 1.70 V at 10 mA cm^−2^	53
N-doped CoP/CeO_2_ on carbon	1.0 M KOH	215 @ 10 mA cm^−2^	74 @ 10 mA cm^−2^	1.52 V at 10 mA cm^−2^	54
CeO_2_/FeS_2_ embedded in N-doped carbon	1.0 M KOH	340 @ 100 mA cm^−2^	—	High activity	55
CeO_2_-Ir/CNTs	0.5 M H_2_SO_4_	≈263 @ 10 mA cm^−2^	—	60 h stable	56

Furthermore, the electrical characteristics of the materials were greatly impacted by defects in the CeO_2_ structure, including oxygen vacancies and structural deformations, and this further illustrated their effect on electrocatalytic performance. Within the bandgap of the material, oxygen vacancies produce discrete electronic structures that can restrict or promote the transfer of electrons. Through serving as active spaces for catalytic reactions, such as the OER, these energy levels change the electronic band structure, the redox behavior of the material, and electrocatalytic activity.

Energy levels and band structures were extremely close to the imperfections impacted by stress and electrical structural modifications brought about by lattice irregularities within the CeO_2_ structure. During electrochemical processes such as the OER, discrete electronic states or changes in the density of states occur that affect the movement of charge carriers and tractions involving immobilized substances. Vacancies within the CeO_2_ lattice can also alter the redox characteristics of the material. Sustainable redox processes combining Ce^3+^ and Ce^4+^ species are made possible by vacancies in oxygen, that serve as electron contributors or acceptors. Considering the number of e^−^ and H^+^ transferred during the OER, this redox process is especially important. Defects in the CeO_2_ structure consequently altered redox potential and kinetics and this, in turn, increased electrocatalytic efficiency. Therefore, the enhanced electrocatalytic activity of CeO_2_/rGO nanostructures in the OER context was a result of the synergistic impacts of intrinsic defects, reduced charge transfer obstructions, and easy electrolyte introduction. Fig. 12c and d display the LSV of the electrocatalyst containing CeO_2_/rGO nanostructures as-prepared and following a 100-h stability test. Chronopotentiometry was also carried out to check the stability of the CeO_2_/rGO nanostructures. At current densities of 10 and 20 mA cm^−2^, they were subjected to an *E*_app_ of 1.5 V for 100 h. Results revealed that the prepared CeO_2_/rGO nanostructure was highly stable and exhibited potential OER ability than the other materials.^57^

### 3.11 Overall water-splitting reaction

**Fig. 13:**
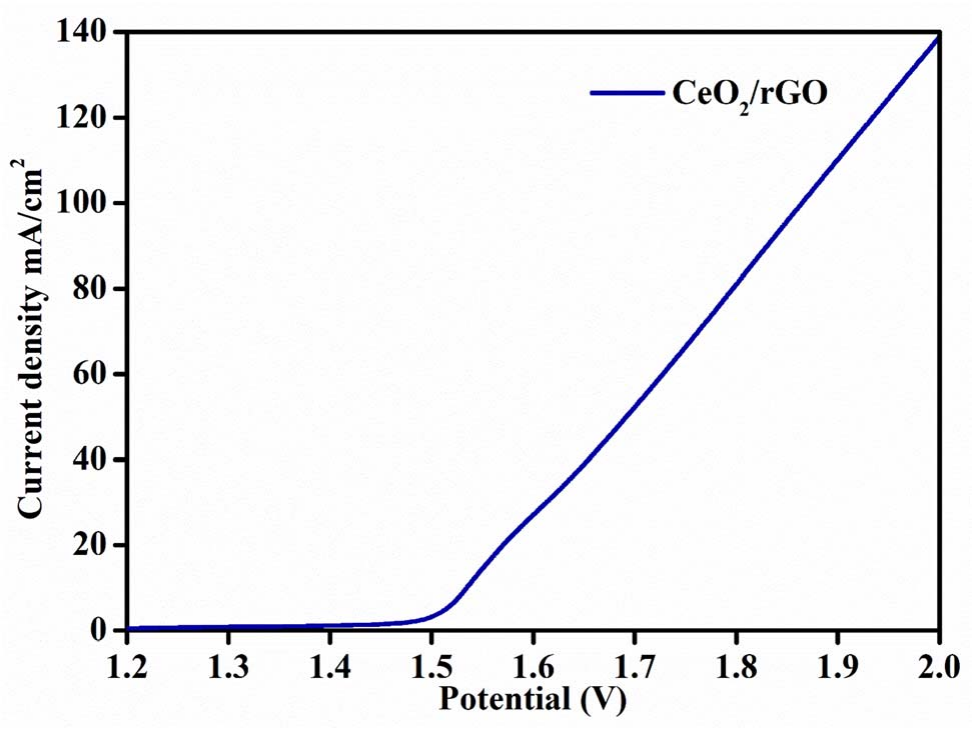
Polarization curve for the overall water-splitting reaction.

The overall water-splitting of the CeO_2_/rGO nanostructures (as an anode and cathode) for the HER and OER involved a two-electrode cell assembly. The bifunctional catalytic action of the nanostructure was measured by LSV within a range of 1–2 V in 1 M KOH (Fig. 13). The catalyst attained 1.52 V cell voltage to reach 10 mA cm^−2^ of current density. The synergetic action between CeO_2_ and rGO in the nanohybrid had a major role in water-splitting. The heterointerface between CeO_2_ and rGO influenced electron-transport facilitates, increased interfacial charge transfer and enhanced hydrogen and oxygen evolution.

### 3.12 EPR spectral analyses

**Fig. 14:**
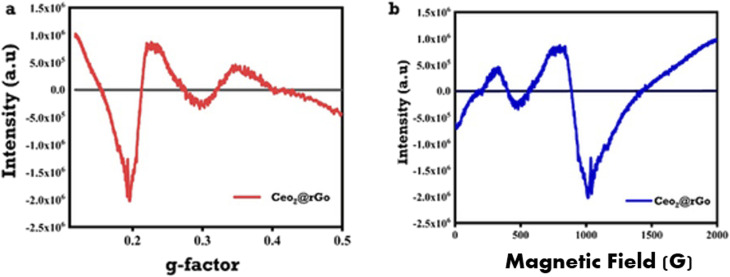
EPR spectra of CeO_2_@rGO nanostructures. (a) g-value. (b) Room temperature with 100-kHz modulation.

The paramagnetic defect centers of the CeO_2_@rGO nanocomposite were characterized using electron paramagnetic resonance (EPR) spectroscopy. Particular concern was paid to oxygen vacancies, which have a major impact on the bifunctional electrocatalytic activity of the material towards the HER and OER. A Bruker EMXplus X-band spectrometer operating at a microwave frequency of 9.857789 GHz was used for measurements, which were carried out at room temperature with 16 scans, a center field of 3480 G, a sweep width of 4000 G, 10 mW of microwave power, and modulation amplitude of 4 G. A strong, asymmetric signal with a significant negative peak near *g* ≈ 0.20–0.22 (with an intensity minimum of roughly −2.0 × 10^6^ to −2.5 × 10^6^ a.u.), a positive lobe near *g* ≈ 0.25–0.30, and weaker oscillatory features extending toward *g* ≈ 0.40–0.50 were present in the CeO_2_@rGO EPR spectrum (Fig. 14). The high signal intensity and characteristic low-*g*-factor signature pointed to a significant concentration of unpaired electrons trapped at oxygen vacancy sites (V–O) within the CeO_2_ lattice. These electrons are often linked to Ce^3+^ (4f^1^) species and are further enhanced by electronic coupling with defect states in the rGO support.^58,59^

The spectrum showed strong derivative-like characteristics in the magnetic field domain (Fig. 14), with zero crossings between ∼500 G and ∼1300–1500 G, consistent with the 100-kHz field modulation, and a significant trough between ∼900 and 1100 G, which corresponded to the highest negative intensity.^60^ The rGO interface encouraged oxygen vacancy formation during synthesis, generating many active sites and enhancing charge carrier mobility, as seen by the increased paramagnetic response in CeO_2_@rGO compared with that of pristine CeO_2_).^61,62^ These defect-induced electronic changes contribute to the observed improved stability, decreased overpotentials, and higher bifunctional performance in alkaline electrolytes by lowering the energy barriers for the adsorption and transformation of HER/OER intermediates (H*, OH*, O*). Hence, EPR gave direct spectroscopic evidence that integrating rGO into oxygen vacancies is an important way to improve CeO_2_-based materials such that they work well as bifunctional electrocatalysts for splitting water.^63^

### 3.13 Proposed charge transfer mechanism at the CeO_2_–rGO interface

**Fig. 15:**
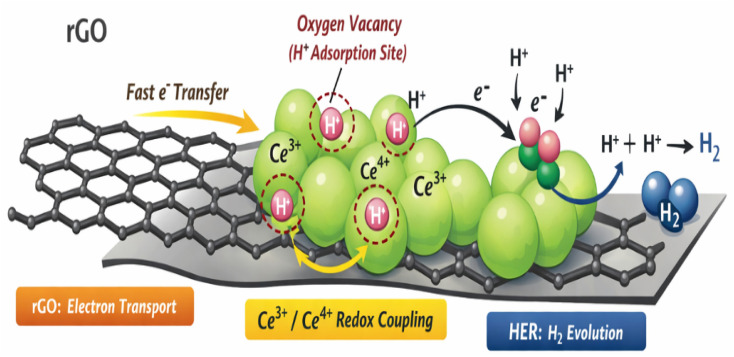
Possible charge transfer and HER mechanism of CeO_2_– rGO.

Fig. 15 shows the possible charge transfer and HER mechanism of CeO_2_–rGO. The superior HER performance of the CeO_2_/rGO hybrid arises from synergistic interfacial electronic coupling and oxygen vacancy engineering. The highly conductive rGO framework acts as an electron transport highway, accelerating charge transfer from the external circuit to CeO_2_ catalytic sites, thereby reducing interfacial resistance.^64^ Strong electronic interaction at the heterointerface modulates the Ce^3+^/Ce^4+^ ratio, promoting charge redistribution and stabilizing oxygen vacancies.^65^ These oxygen vacancies provide energetically favourable H^+^ adsorption sites due to their unsaturated coordination and localized electron density.

During the HER in alkaline media, water dissociation and proton adsorption occur at vacancy sites (Volmer step), followed by electron transfer mediated through rGO. The reversible Ce^3+^/Ce^4+^ redox couple facilitates intermediate stabilization and lowers the kinetic barrier for hydrogen evolution.^66^ Subsequent hydrogen formation proceeds *via* Heyrovsky or Tafel pathways. Thus, enhanced conductivity, abundant defect sites, and redox flexibility collectively improve catalytic efficiency.

## 4 Conclusions

A hybrid nanostructured electrocatalyst was synthesised and systematically examined for bifunctional water electrolysis. XRD and Raman analyses of the structure and phases confirmed that a well-integrated hybrid framework had formed with no detectable impurity phases. FESEM and TEM images showed a uniform nanoscale morphology with close interfacial contact between different parts. XPS analysis confirmed the chemical states and strong electronic interaction between elements, which is good for electrochemical activity. Electrochemical studies showed that the catalyst worked very well for the HER and OER in alkaline media. For the HER, the catalyst needed a low overpotential of ∼260 mV to reach −50 mA cm^−2^ and 350 mV to reach −100 mA cm^−2^ For the OER, the overpotential was ∼230 mV at 10 mA cm^−2^. Tafel slopes were not very steep, so the reaction kinetics were good. EIS further validated diminished charge-transfer resistance, emphasising effective electron transport at the electrode–electrolyte interface. This better performance was due to the way the hybrid parts worked together, the larger electrochemically active surface area, and better electrical conductivity. Most importantly, the ability of the catalyst to work in two ways was tested in a two-electrode electrolyser setup for splitting water. The assembled electrolyser produced a low cell voltage that was comparable with the performance of recently reported bi-functional electrocatalysts. Also, long-term durability tests showed that the current density and cell voltage did not change much over 100 h of continuous use, which suggested that the device was very stable during use. Overall, this study shows a clear relationship between structure, property, and performance. It also shows that the new hybrid nanostructure is a promising bi-functional electrocatalyst for stable and efficient water electrolysis. The results offer important design insights for the development of next-generation, economical electrocatalysts intended for sustainable hydrogen production.

## Conflicts of interest

There are no conflicts to declare.

## Supplementary Material

RA-016-D5RA09363E-s001

## Data Availability

Data will be made available upon request. Supplementary information (SI) is available. See DOI: https://doi.org/10.1039/d5ra09363e.
