# Supplementary material for: High-performance CeO2/rGO hybrid nanostructures as bifunctional electrocatalysts for water-splitting
Source: RSC Adv. 2026 May 1;16(25):22861–78. doi: 10.1039/d5ra09363e (PMC13133710; doi:10.1039/d5ra09363e)
Supplement: RA-016-D5RA09363E-s001 [file RA-016-D5RA09363E-s001.pdf]

Supplementary Information (SI) for RSC Advances.
This journal is © The Royal Society of Chemistry 2026

High-Performance CeO_2/rGO Hybrid Nanostructures as Bifunctional Electrocatalysts for Water Splitting

John Andrews Staline.C [1] , C. Maria Magdalane [2] , Gopal Ramalingam [3] , Jaya Dwivedi [1*] , Saikh Mohammad Wabaidur [4]

[1] Department of Chemistry, Banasthali Vidyapith, Rajasthan-304022, India.

[2] Department of Chemistry, St. Xavier's College (Autonomous), Palayamkottai, Tirunelveli 627002, (Affiliated to Manonmaniam Sundaranar University, Abishekapatti, Tirunelveli-627012)

[3] Quantum Materials Research Lab (QMRL), Department of Nanoscience and Technology, Alagappa University, Karaikudi-630003.

[4] Chemistry Department, College of Science, King Saud University, Riyadh 11451, Saudi Arabia.

*Corresponding Author: E-mail Address: jayadwivedi@yahoo.co.in (Jaya Dwivedi)

3.8.1 XPS Spectrum of Reduced graphene oxide (rGO)

The X-ray photoelectron spectroscopy (XPS) analysis of reduced graphene oxide (rGO) reveals its elemental composition and surface functional groups. The survey spectrum shows dominant peaks for C1s (~284 eV) and O1s (~531 eV), indicating that carbon predominates with reduced oxygen post-reduction. The decreased O1s intensity suggests effective removal of oxygen-containing groups. The high-resolution C1s spectrum identifies peaks for sp^2 hybridized carbon (C–C/C=C) at ~284.5 eV and residual oxygen functionalities such as C–O, C=O, and O–C=O, reflecting partial restoration of the conjugated carbon network. The O1s spectrum shows peaks for C=O, C–OH, and C–O–C, with lower intensity indicating removal of most oxygen functionalities, although some remain. Overall, the XPS results corroborate the successful reduction of graphene oxide into rGO, marked by high carbon content, diminished oxygen functionalities, and restoration of the sp^2 network, which benefits electrical conductivity and material performance in various applications.

XPS

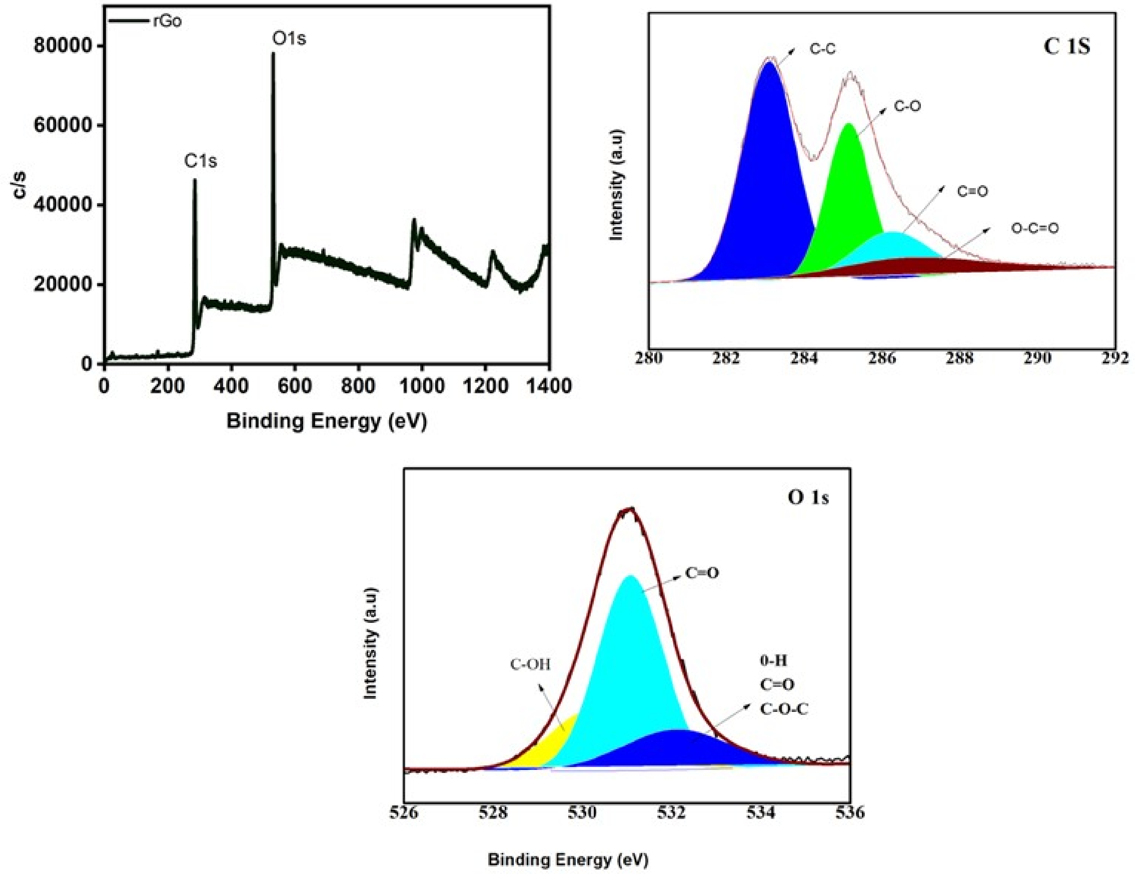

Figure S8.1: XPS analysis of rGO nanostructure

3.8.2 1 XPS Spectrum of CeO_2

A comprehensive examination of the Ce 3d spectrum, characterized by complexity, allows for deconvolution into multiple peaks reflecting the spin–orbit components Ce $3d_{5/2}$ and Ce $3d_{3/2}$. These peaks are further divided into contributions from Ce^{3+} and Ce^{4+} oxidation states, with Ce^{4+} peaks indicating successful CeO_2 formation, alongside additional peaks for Ce^{3+} signifying oxygen vacancies or defects within the crystalline lattice. The coexistence of Ce^{3+}/Ce^{4+} redox pairs is crucial, enhancing the catalytic and biological effectiveness of CeO_2. In the high-resolution O 1s spectrum, two primary components emerge: a major peak at approximately 529–530 eV corresponds to lattice oxygen (O^{2-}) in the CeO_2 structure, while a secondary peak at 531–532 eV relates to surface-adsorbed oxygen species, hydroxyl groups, or defect-associated oxygen. The intensity of the higher binding energy peak corroborates the existence

of oxygen vacancies and surface functionalities. In conclusion, the XPS findings affirm the successful synthesis of CeO_2 nanoparticles characterized by mixed valence states (Ce^{3+}/Ce^{4+}) and oxygen vacancies, which are essential for enhancing the material's redox properties, catalytic performance, and its potential roles in biomedical and environmental applications.

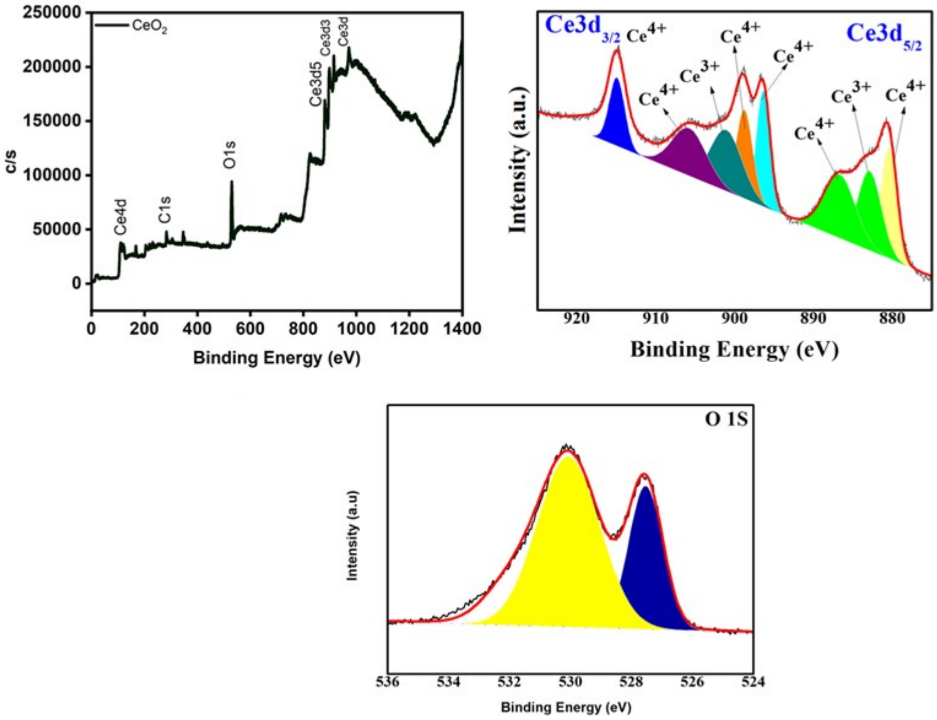

Figure S8.2 : XPS analysis of CeO_2 nanostructure
